# Hybrid Membrane Technology for Acid Recovery from Wastewater in Coated Steel Wire Production: A Pilot Scale Study

**DOI:** 10.3390/membranes12121196

**Published:** 2022-11-27

**Authors:** Sergey Loza, Natalia Loza, Alexander Korzhov, Nazar Romanyuk, Nikita Kovalchuk, Stanislav Melnikov

**Affiliations:** Physical Chemistry Department, Faculty of Chemistry and High Technologies, Kuban State University, 350040 Krasnodar, Russia

**Keywords:** diffusion dialysis, concentration, electrodialysis, ion-exchange membrane, separation of acids and salts, acid recovery

## Abstract

In the present study, the problem of sulfuric acid recycling from spent copper plating solution was solved using a hybrid membrane technology, including diffusion dialysis and electrodialysis. A real solution from the production of copper-coated steel wire, containing 1.45 mol/L of sulfuric acid, 0.67 mol/L of ferrous sulfate and 0.176 mol/L of copper sulfate, was processed. Diffusion dialysis with anion-exchange membranes was used to separate sulfuric acid and salts of heavy metals. Then, purified dilute sulfuric acid was concentrated by electrodialysis. The energy consumption for sulfuric acid electrodialysis concentration at a current density of 400 A/m^2^ was 162 W·h/mol, with a current efficiency of 16%. After processing according to the hybrid membrane scheme, the solution contained 1.13 mol/L sulfuric acid, 0.077 mol/L ferrous sulfate and 0.022 mol/L copper sulfate. According to established requirements, the solution of a copper plating bath had to contain from 0.75 to 1.25 M sulfuric acid, 0.16–0.18 M of copper sulfate and ferrous sulfate not more than 0.15 M. The resulting acid solution with a small amount of ferrous sulfate and copper sulfate could be used to prepare a copper plating bath solution.

## 1. Introduction

The gradual exhaustion of natural resources requires developing non-waste technologies, allowing the rational expenditure of raw material. The increasing volume of liquid waste containing mineral acids is connected with the dynamic development of industry in the last few years. Mineral acids are widely used in the chemical industry and hydrometallurgy, in the processes of metal etching and galvanic coating [1]. With a decrease in the concentration of acid or its contamination with impurities of a certain concentration, the solution turns into waste. The spent acid containing various heavy metal ions possesses both a high corrosion activity and toxicity. Such waste materials might do irreversible harm to the environment. Precipitation of heavy metals with lime and alkali is the traditional and most often used method of neutralization, such as with wastewaters [2]. However, the acids are irretrievably lost, and hazardous solid waste emerges in the process. Leaching of heavy metals into natural waters creates serious ecological damage [3,4]. The membrane methods allow for solving such problems as the return of valuable resources to the production and the establishment of zero liquid discharge plants, leading to a decrease in environmental pressures [5,6,7]. Membrane filtration processes and dialysis are the most common ones [8,9]. Electrodialysis allows for separating and concentrating the components of various technological solutions and wastewaters containing electrolytes [10,11], but its usage is limited because of the cumbersomeness of the instrumental design and high energy consumption. In the case of electrodialysis concentration, this technology appears to be competitive, because the alternatives, such as vaporizing, are even more energy consuming [12]. The problem of mixed electrolyte solutions separation is still highly relevant, despite the appearance of many manuscripts dedicated to developing membranes with specific selectivity in the processes of electromembrane separation. Either separation coefficients of ions of the same charge sign have low values, or used membranes are experimental prototypes [13,14,15,16]. Furthermore, when the operating current density is higher than the limiting one, the specific permselectivity of such membranes decreases significantly. Hence, the practical relevance of using these processes in the industry is low [17]. In this regard, the dialysis method of separating acids and heavy metal salts using ion-exchange membranes is promising, because it is not energy-consuming. A number of papers have shown the efficiency of the separation of salts and acids by diffusion dialysis with anion-exchange membranes [18,19,20]. It has been shown in [21,22] that the most promising in terms of energy and water consumption is a combination of diffusion dialysis and electrodialysis methods for a more complete recovery of mineral acids.

This paper proposes a hybrid membrane technology for the recovery of sulfuric acid from a waste solution, which is formed in a workshop for the production of copper-coated steel wire. In the process of wire manufacturing, at the stage of its pull-out through dies, a lubricant based on metal stearates is used to reduce the friction coefficient. After obtaining the wire of the required diameter, the lubricant is removed in a bath with heated phosphoric acid under the ultrasonic action. The wire then passes through a bath of sulfuric acid and copper sulfate.

In this bath, iron is oxidized to Fe^2+^ ions with simultaneous reduction of Cu^2+^ ions to metallic copper, which is deposited on the wire surface. When the concentration of Fe^2+^ ions exceeds approximately 1 mol/L, the solution is disposed of and replaced with a fresh one. At present, the reagent method of lime precipitation is used for the disposal of the solution. At the same time, about 1800 kg of sulfuric acid and 940 kg of copper sulphate per month are irretrievably lost, and a large amount of solid waste is generated. The development of sulfuric acid recovery technology will lead to a reduction in the amount of solid waste.

**Figure 1:**
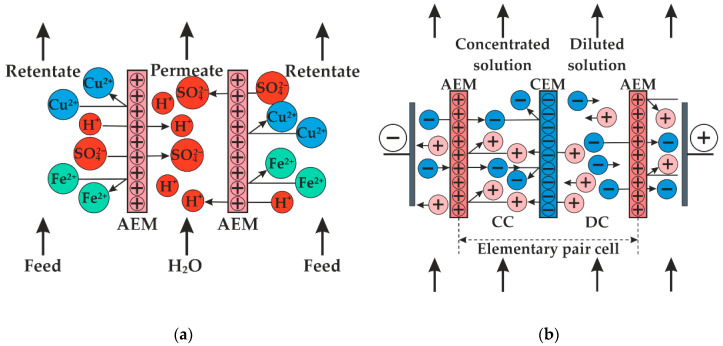
Scheme of ion fluxes in the diffusion dialysis (**a**) and electrodialysis (**b**) processes. Where DC is the desalination chamber and CC is the concentration chamber.

**Figure A1:**
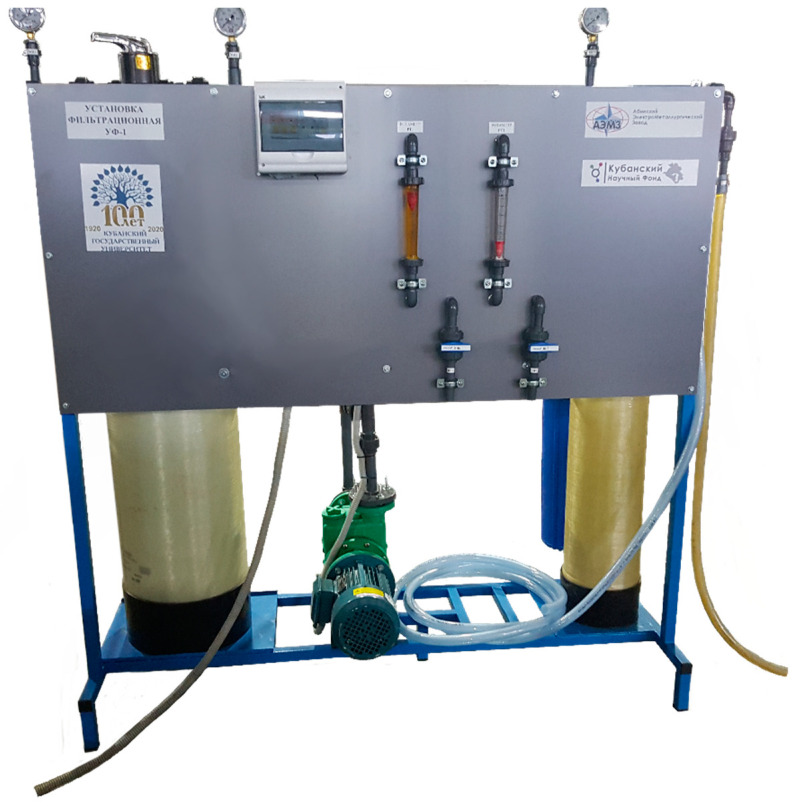
The photo of the filtration module.

To solve this problem, a hybrid membrane technology is proposed, including solution processing with diffusion dialysis and electrodialysis. Before this procedure, it has to be cleaned from stearates and scales. To do so, the pre-treatment stage in the filtration module using granular filtration is included in the solution processing scheme. The appearance of the filtration module is shown in Figure A1 in Appendix A. After the filtration module, sulfuric acid is isolated from the solution by diffusion dialysis using anion-exchange membranes. The scheme of the separation process of salts and sulfuric acid is shown in Figure 1a. The mixed solution of acid and salts flows on one side of the anion-exchange membrane, and water flows on the other one. As a result, a concentration difference is created, causing a diffusion flux of dissolved electrolytes from solution into water through the membrane. Since the anion-exchange membrane contains positively charged fixed groups, diffusion of cations across the membrane is hindered as a result of electrostatic repulsion forces. Cations that have a lower charge and higher mobility are transferred first. In this case, these are hydrogen cations, which in general determine the rate of diffusion transfer [20,23,24]. Thus, the dialysis module separates sulfuric acid, which is transported through the anion-exchange membrane from copper and ferrous sulfates, which remain in the processed solution.

The papers [25,26] describe a scheme for the regeneration of sulfuric and hydrochloric acids using spiral diffusion dialysis modules with a counter-current flow. Such spiral modules have a high cost and a non-separable design, which requires careful pretreatment of solutions. In addition, the purification of solutions occurs in one pass, and the concentration gradient over the entire length of the module is low. This leads to a low speed of operation of such modules. The use of a circulation scheme with regular dialysate renewal allows for maintaining a high concentration gradient and accelerating the removal of acid from the processed solution. However, this forms a large amount of dilute acid solution unsuitable for industrial use. This problem can be solved by concentrating the acid solution to the level required by the technical regulations.

Electrodialysis is an effective method of concentrating electrolyte solutions. According to IUPAC terminology, electrodialysis is the membrane-based separation process in which ions are driven through an ion-selective membrane under the influence of an electric field [27]. The electrodialysis apparatus consists of alternating cation-exchange (CEM) and anion-exchange (AEM) membranes that form the concentration and the desalination chambers. The chamber of concentration and desalination form an elementary pair cell. Figure 1b shows the scheme of ion transport in the elementary pair cell of the electrodialysis apparatus. Under the action of an electric field, protons are transferred through the cation-exchange membrane, and sulfate anions are transferred through the anion-exchange membrane into the concentration chamber. Thus, the concentration of acid in it increases, while in the desalination chamber it decreases.

Successful laboratory scale studies of the separation of salts and acids by dialysis or electrodialysis are described, and a very limited number of works are devoted to pilot or industrial scale ones [9,28,29]. A pilot scale study of the membrane utilization of the acidic waste, including the electrodialysis concentration of the diluted permeate solution, is the novelty of present work.

In the present work, a hybrid technology, including the separation of sulfuric acid and sulfates of Cu^2+^ and Fe^2+^ by dialysis and the subsequent concentration of the extracted acid by electrodialysis, has been used to process a real technological solution for a wire production workshop to assess the possibility of its application in production conditions. Obviously, the rate of substance transfer in electrodialysis is significantly higher than in dialysis due to the difference in their driving forces. Therefore, for the successful application of the proposed technology, it is necessary to determine the optimal operating modes of the dialyzer and electrodialyzer.

## 2. Materials and Methods

### 2.1. Membranes

The Ralex CMHPES and Ralex AMHPES membranes (Mega a.s., Prague, Czech Republic) were used as cation-exchange and anion-exchange membranes in the electrodialysis (ED) module. The Ralex AMHPES membranes were used in the dialysis module. These IEMs are heterogeneous membranes, which consist of ion-exchange resins, polyethylene and polyester mesh. Ion-exchange resins are co-polymers of styrene and divinylbenzene with sulfonic acid ionogenic groups in the case of cation-exchange resin and quaternary ammonium bases in the case of anion-exchange resin. The main physical-chemical properties of the IEMs are presented in Table 1. The ion-exchange capacity (*Q*) was determined for H^+^- and OH^−^-form of cation- and anion-exchange membranes, respectively. The Ralex CMHPES and Ralex AMHPES membranes were immersed in solutions containing the excess of NaOH or HCl, respectively. OH^−^ or H^+^ ion concentration in studied solutions was determined by acid–base titration after 24 h, and the value of *Q* was calculated as follows

(1) $$Q=\frac{V\Delta C}{m_{wet}},$$

where *V* was the studied solution volume, Δ*C* was the difference between the initial and final OH^−^-ions and H^+^-ions concentration in the studied solution for the Ralex CMHPES and Ralex AMHPES membranes, respectively, and *m_wet_* was the mass of the wet membrane. The membrane thickness (*l*) was in H^+^- and ${\mathrm{SO}}_{4}^{2-}$-ion forms.

### 2.2. Waste Characteristics

The composition of waste water is shown in Table 2. The content of ferrous sulfate in the acid solution should be no more than 0.15 M for reuse of the acid in the copper plating bath.

### 2.3. Pilot Scale Hybrid Installation

**Figure 2:**
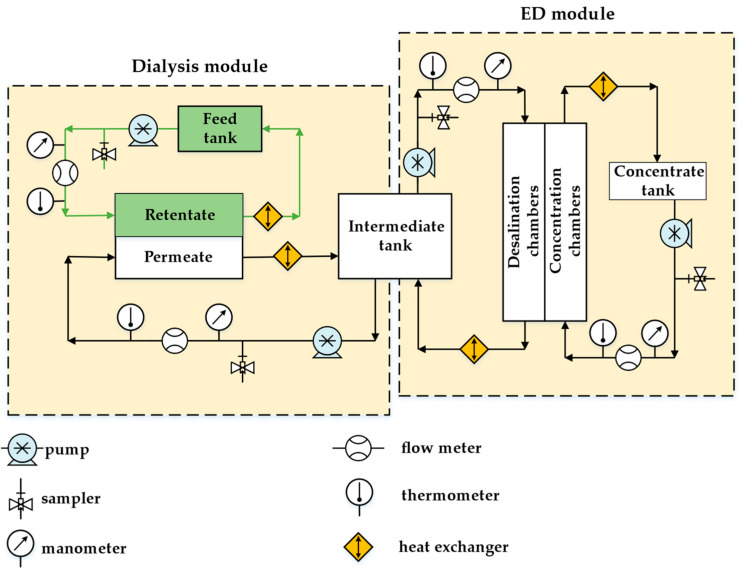
Simplified scheme of the pilot scale installation.

Figure 2 shows the scheme of the pilot scale hybrid installation. The main modules were the dialysis and the electrodialysis ones. Both membrane modules operated in a cyclic mode. The feed solution and the permeate circulated through the dialysis module in a co-current flow regime. The sulfuric acid penetrated through the AEMs in the dialysis module, and the salts remained in the retentate. The permeate contained mostly sulfuric acid and a small amount of salts. The permeate was supplied from the dialysis module to the intermediate tank. The intermediate tank was used as a feed tank for the desalination chambers of the ED module, where the sulfuric acid was concentrated.

**Figure 3:**
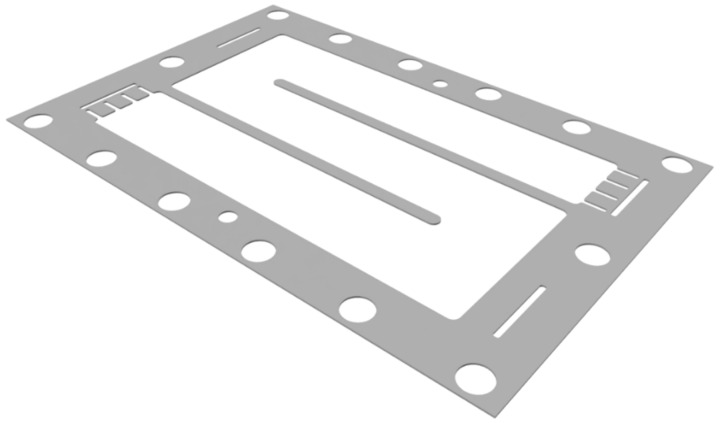
The image of the gaskets of the “labyrinth” shape for the dialysis module.

**Figure A2:**
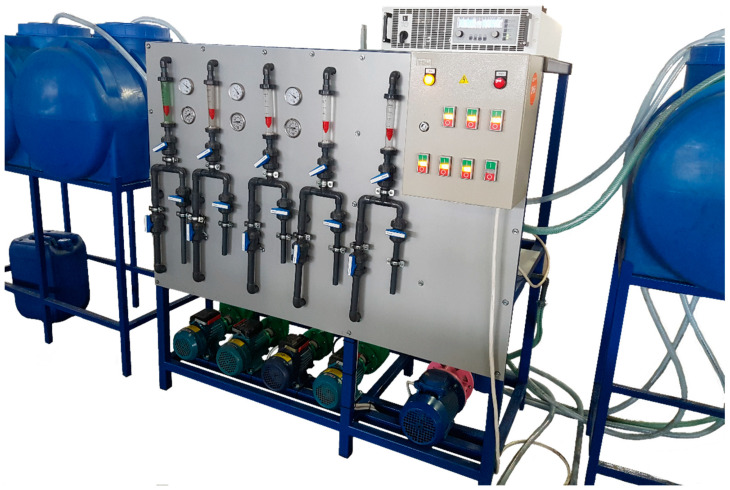
The photo of the pilot scale hybrid installation.

The dialysis and ED modules of our own production (in the scientific and technological park, “Technopark University”, of Kuban State University, Krasnodar, Russia) were used. The appearance of the pilot scale hybrid installation is shown in Figure A2 in Appendix A. The dialysis module consisted of 50 AEMs. The membrane size was 42 × 28 cm^2^, the working membrane area was 0.06 m^2^ and the total membranes area was 3 m^2^. The AEMs were separated by gaskets. The distance between the membranes was 0.9 mm. The gaskets of special “labyrinth” shape (Figure 3) made it possible to increase the length of the solution path and liner velocity in the dialysis module and the efficiency of the process in one pass. The solution linear velocity through chambers was 0.025 m/s at the pressure 10 kPa.

The ED module included 25 elementary pair cells, each one consisting of the cation- and anion-exchange membranes. The cation-exchange membrane was Ralex CMHPES, and the anion-exchange membrane was Ralex AMHPES. There were 25 Ralex CMHPES membranes and 26 Ralex AMHPES membranes in the ED module. The working membrane size was 0.1 × 0.4 m^2^. The distance between the membranes in the electrodialysis unit was 0.9 mm, and the membranes were separated by the polyethylene gaskets. Inert nylon mesh spacers were placed between membranes. The solution linear velocity through chambers was 0.015 m/s at pressure 30 kPa. The working mode was galvanostatic, and the current density was fixed at 400 A/m^2^.

The intermediate tank solution fed the desalination chambers, and the dilute sulfuric acid fed the concentration chambers at the first stage. The concentrate solution obtained at the first stage was used as a feed solution for the concentration chamber at the second stage. The volume and composition of the solution in the feed, intermediate and concentrate tanks were determined at certain intervals. Based on the experimental data, the main characteristics of the process of dialysis separation and electrodialysis concentration were calculated according to Equations (2)–(4)

(2) $$j_{i}=\frac{C_{i}^{t}V^{t}-C_{i}^{t-1}V^{t-1}}{\Delta tnS},$$

(3) $$\eta_{i}=\frac{(C_{{}_{i}}^{t}V^{t}-C_{{}_{i}}^{t-1}V^{t-1})zF}{nI\Delta t},$$

(4) $$W=\frac{I\int_{0}^{t}Udt}{\nu_{t}},$$

where *J_i_* is integral flux of *i* electrolyte; $C_{i}^{t}$ is the concentration of *i* electrolyte at time *t*; $V^{t}$ is the volume of the solution in feed tank for dialysis and concentrate tank, respectively; n is number of elementary cells in the membrane unit; *S* is the working area of membrane; *t* is the time of experiment; *η* is a current efficiency; z is a charge number of sulfate anion; *F* is Faraday constant; *I* is current intensity; *W* is energy consumption for obtaining 1 mole of a substance; *U* is the voltage applied on the electrodialysis unit and *ν_t_* is the amount of electrolyte transferred to concentrate tank during time *t*.

### 2.4. Method of Determining the Composition of Solutions Containing Acid and Metal Salts

The H_2_SO_4_ concentration in the solutions was determined by potentiometric titration using the EasyPlus Automated Titrator (N.V. Mettler-Toledo S.A., Zaventem, Belgium).

The concentration of the Cu^2+^ ions in the solutions was determined by the Red-Ox titration. An excess of 20% solution of KI was added to an aliquot of the analyzed solution and left for 5 min in the dark. After that, the test solution was titrated with 0.1 mol-eq/L Na_2_S_2_O_3_ solution until the color of the solution changed from dark brown to pale yellow (straw). Then, 5–6 drops of 0.1% starch solution were added to the test solution and the titration was continued until the color of the solution changed from blue to pale green.

The concentration of the Fe^2+^ ions in the solutions was determined by titration with the KMnO_4_ solution. Added to an aliquot of the analyzed solution was 5–10 mL of 85% orthophosphoric acid to bind Fe^3+^ ions.

The procedure of each titration was repeated at least three times. The result was taken as the arithmetic mean of all determinations.

## 3. Results

### 3.1. Acid Recovery from Wastewater of the Coated Steel Wire Production. Stage 1

**Figure 4:**
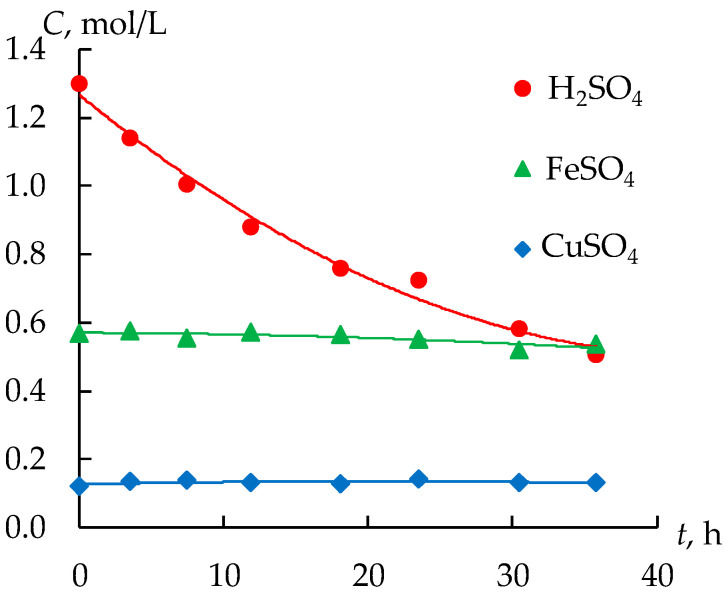
Dependence of the change in the concentrations of components in the feed tank on the time of dialysis.

At the beginning of the experiment, 0.15 M sulfuric acid is used to fill the concentrate tank of the electrodialysis module, and tap water is used to fill the intermediate tank. A dilute sulfuric acid solution is used to fill the concentration tank to ensure good electrical conductivity, which is necessary to start the electrodialysis concentration process. The first stage of the experiment lasts about 36 h, until a constant concentration of sulfuric acid is reached in the concentration tank. Figure 4 shows the change of the concentration of all the components in the retentate. As expected, the concentration of sulfuric acid reduces in the dialysis by about three times from 1.3 mol/L to 0.5 mol/L. At the same time, concentrations of the copper sulfate and the ferrous sulfate (II) do not practically change. Thus, diffusion dialysis makes it possible to successfully separate acid and salts.

**Figure 5:**
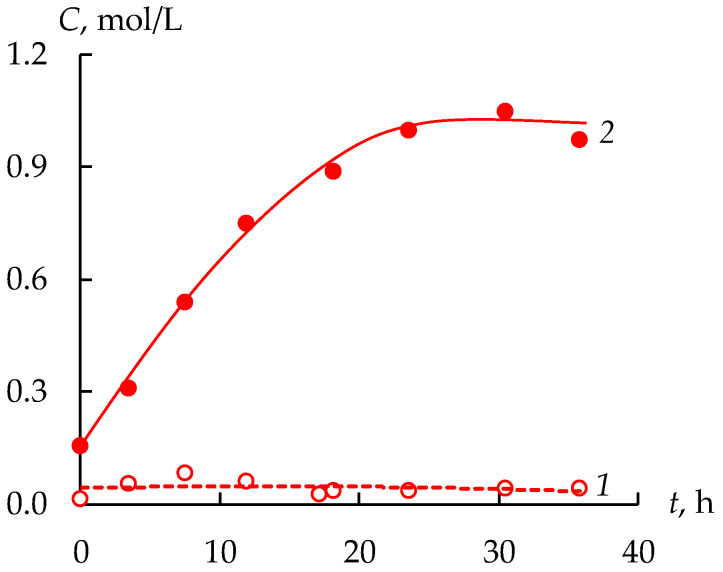
The sulfuric acid concentrations in the intermediate (1) and concentrate (2) tanks during the operation of the installation.

The permeate circulates through an intermediate tank from which the desalination chambers of the ED module are fed. The concentration of the sulfuric acid in the desalination chambers of the ED module is reduced because of the transfer of cations through CEMs and anions through AEMs. It leads to a gradual increase in sulfuric acid concentration in the concentration chambers (Figure 5). The sulfuric acid concentration in the concentration tank increases during the first 20 h from 0.15 M to 1 M, and after that it practically stops changing. Despite the fact that the concentration of sulfuric acid decreases when the solution passes through the desalination chamber, its concentration is remains almost constant in the intermediate tank due to the operation of the dialysis module.

**Figure 6:**
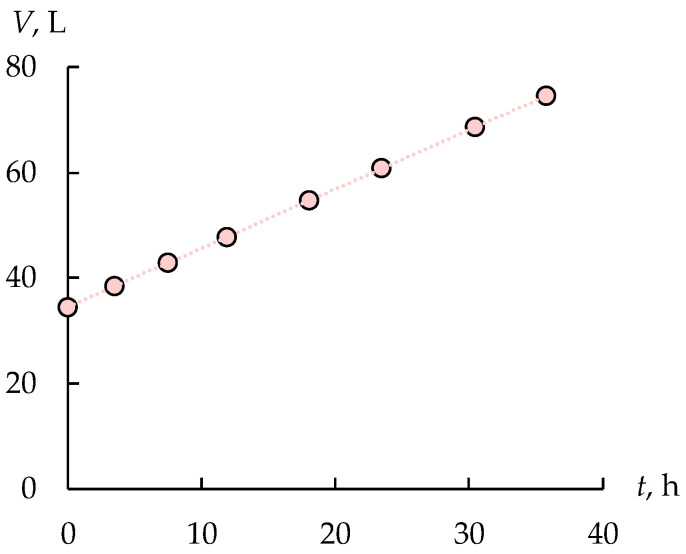
The solution volume in the concentrate tank during the first stage.

The sulfuric acid concentration remains constant in the concentration tank due to the electroosmosis and osmosis water transfer and the diffusion flux of electrolytes, which occurs due to the difference in the concentrations between the concentration and the desalination chambers. The increase in the solution volume in the concentrate tank by two times confirms the presence of significant water transfer (Figure 6). The electroosmosis and osmosis water transfer is the reason for limiting the maximum achievable solution concentration [30,31]. The change in the solution volume in the feed tank does not exceed the experimental error at the same time.

The intermediate solution and the concentrate solution, which is obtained in the first stage, are used in the second stage. Thus, the diluted sulfuric acid is used as intermediate solution instead of tap water, and the 1 M sulfuric acid solution is used as the concentrate solution instead of the 0.15 M sulfuric acid solution.

The operating mode of the ED module has been also chosen in the first stage, which ensures a constant concentration of acid in the intermediate tank. The presence of a sufficient amount of acid in the intermediate tank is a necessary condition for keeping energy consumption for desalting in the ED module at a low level. If the acid concentration decreases greatly in the intermediate tank, the voltage on the ED module increases. It leads to an increase in energy consumption. There are two ways to solve this problem. The first option is the periodic operation of the electrodialysis module, which will turn on only for certain periods of time, when the acid concentration in the intermediate tank is sufficient, to ensure its operation.

The disadvantage is the change in the concentration of the solution in the chambers of the electrodialyzer due to the osmotic flow of water, which is directed from the desalination chambers to the concentration chambers, as well as the diffusion flow of acid in the opposite direction. Both of these streams result in a decrease in acid concentration. In this regard, the continuous mode of operation of the ED module seems to be more promising, and it will be synchronized with the operation of the dialysis module so that the dialysis module has time to supply enough acid to the intermediate tank to ensure the operation of the ED module. It has turned out that the galvanostatic mode with a current strength of 16 A (400 A/m^2^) is the most suitable.

The optimal operating mode of ED module has been determined. The sulfuric acid concentration in the concentration tank is increased from 0.15 to 1 mol/L in the first stage.

### 3.2. Acid Recovery from Wastewater of the Coated Steel Wire Production. Stage 2

**Figure 7:**
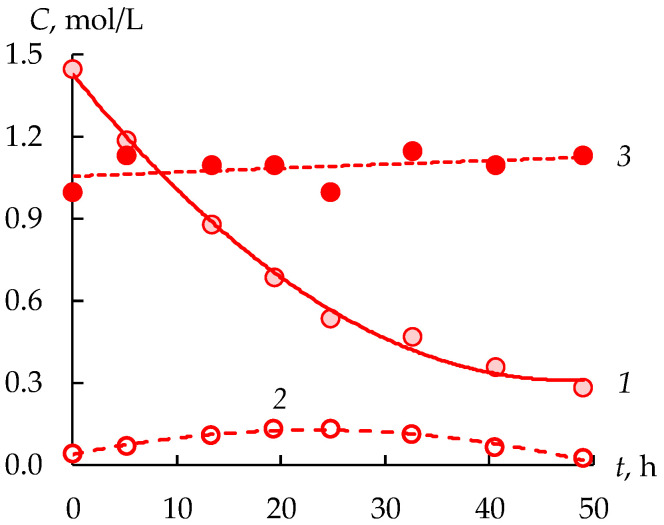
Sulfuric acid concentrations in the feed (1), intermediate (2) and concentrate (3) tanks during the second stage.

The sulfuric acid concentration in the concentrate tank increases from 1.00 to 1.13 mol/L in the stage 2 and decreases in the feed tank to 0.28 mol/L in 50 h (Figure 7). The concentration in the intermediate tank first increases from 0.042 to 0.133, and then decreases to 0.027 mol/L. Obviously, the decrease in the acid concentration is due to the fact that its concentration in the electrodialysis module occurs somewhat faster than the accumulation as a result of dialysis.

**Figure 8:**
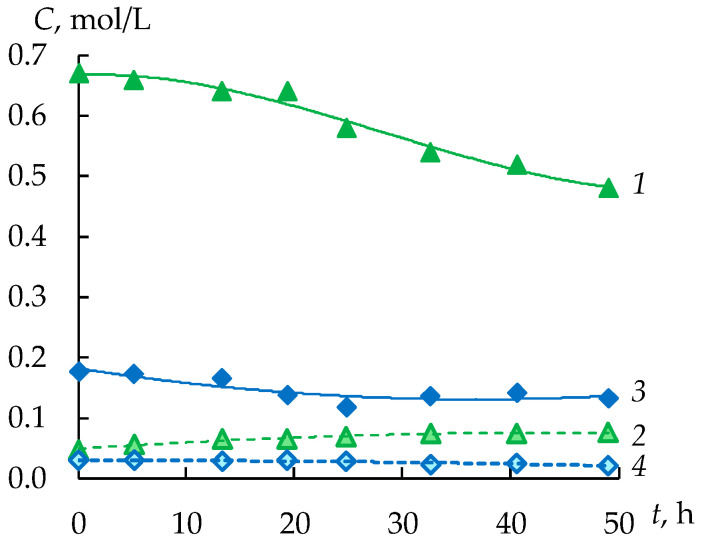
Concentrations of FeSO_4_ (1, 2) and CuSO_4_ (3, 4) in the feed (1, 3) and concentrate (2, 4) tanks during the second stage.

**Figure 9:**
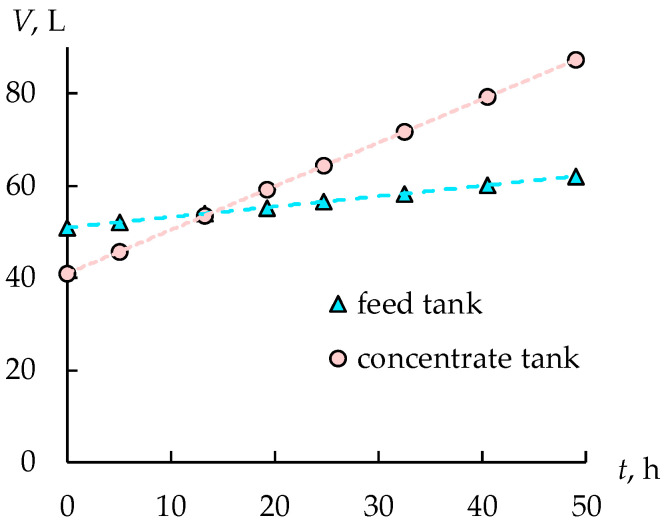
The solution volume in the concentrate and feed tanks during the second stage.

The concentrations of FeSO_4_ and CuSO_4_ are reduced by 28% and 25%, respectively, in the feed tank (Figure 8). At the same time in the concentrate tank, the concentration of ferrous sulfate increases to 0.077 mol/L, and copper sulfate to 0.022 mol/L, which does not exceed the values allowed for the reuse of sulfuric acid. An interesting fact is the decrease in the concentration of copper sulfate during electrodialysis in the concentration tank from 0.030 to 0.022 mol/L. This is due to the low copper sulfate flux, which is 0.01 mol/(m^2^·h). The decrease in the concentration of copper sulfate in the concentrate at a positive value of the flow is due to the dilution of the solution as a result of an increase in the volume of the solution by two times (Figure 9). This is due to the osmotic and electroosmotic transfer of water from the desalination chambers to the concentration chambers of the electrodialyzer. At the same time, the average flow of ferrous sulfate is 10 times higher than the flow of copper sulfate and is 0.097 mol/(m^2^·h), which is explained by its higher concentration in the feed and intermediate tanks.

**Figure 10:**
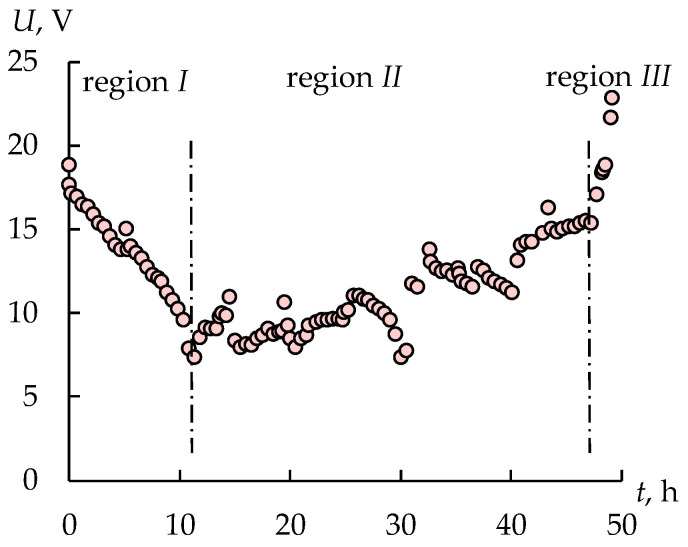
The current density and voltage drop on the electrodialyzer during the second stage.

**Figure 11:**
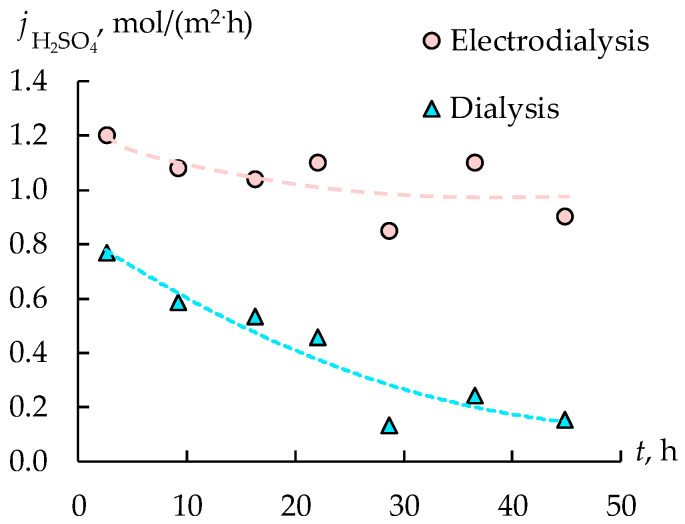
The fluxes of sulfuric acid during dialysis and electrodialysis.

Figure 10 shows the change of a voltage drop value during the electrodialysis concentration of sulfuric acid solution. There are three regions on the *U*-*t* dependence. The first region is the reduction in *U* value due to the increase in the concentration of sulfuric acid in the intermediate tank that feeds the desalination chambers. Increasing the sulfuric acid concentration in the concentrate tank also leads to the decrease in the *U* value. The second region is the gradual increase in *U* value because of the reduction in the sulfuric acid concentration in the intermediate tank. The third region is rapidly rising *U* values that can be explained by the significant decrease in sulfuric acid flux in the dialysis module (Figure 11).

The flux of acid during dialysis decreases over time, which is associated with a decrease in the concentration gradient between retentate and permeate (Figure 11). The flux in the electrodialyzer first decreases slightly, and then practically stops changing. This is due not only to the water flux diluting the solution in the concentration chamber, but also to the reverse diffusion of electrolytes from the concentration chambers to the desalination chambers, as well as to the nonselective migration transfer of hydrogen cations through the anion-exchange membranes from the concentration chamber to the desalination chamber. All these factors limit the maximum achievable degree of concentration.

Based on the data obtained, the average energy consumption for the concentration of sulfuric acid was calculated, amounting to 162 W·h/mol. It is similar or somewhat lower than the results available in other works. Thus, it is shown in [32,33] that for solutions comparable in composition, the energy consumption is 219–430 W·h/mol. It turns out that the current efficiency for sulfuric acid does not exceed 16%. Such a low current efficiency is due to the back diffusion of sulfuric acid, as well as proton leakage through anion exchange membranes [34,35,36].

## 4. Conclusions

A hybrid membrane technology has been developed, which includes dialysis and electrodialysis, making it possible to purify acids from salts of heavy metals and return acids to the technological process in the production of steel wire. It has been determined that during electrodialysis, an increase in the volume of a concentrated sulfuric acid solution by more than two times is observed. This occurs not only as a result of the transfer of acid, but also as a result of the presence of a water flux from the desalination chambers to the concentration ones. However, an increase in the concentration of sulfuric acid from 0.15 mol/L, which is used to fill the concentration chambers at the beginning of the installation operating, to 1.1 mol/L during the operation of the ED module is found. The sulfuric acid recovery as a result of waste treatment using hybrid membrane technology is 78%. During the recovery of sulfuric acid from the waste copper plating solution, regenerated acid is obtained with a concentration of 1.1 mol/L and a residual metal content of less than 0.1 mol/L, which allows it to be reused in the technological process.

## Figures and Tables

**Table 1:** The physical-chemical properties of IEM.

Membrane	*l*, μm	*Q*, mmol/g_wet_
Ralex CMHPES	540	1.16
Ralex AMHPES	550	0.86

**Table 2:** The composition of waste water.

Component	C, mol/L
H_2_SO_4_	1.45
CuSO_4_	0.176
FeSO_4_	0.67

## Data Availability

The data presented in this study are available on request from the corresponding author.
